# The monetary value of human life losses associated with COVID-19 in Turkey

**DOI:** 10.35241/emeraldopenres.13822.1

**Published:** 2020-07-08

**Authors:** Joses Muthuri Kirigia, Rose Nabi Deborah Karimi Muthuri, Lenity Honesty Kainyu Nkanata

**Affiliations:** 1 African Sustainable Development Research Consortium (ASDRC), Nairobi, P.O. Box 6994 00100, Kenya; 2 Faculty of Health Sciences, University of Pretoria, Pretoria, Hatfield, Pretoria, 0002, South Africa

**Keywords:** Coronavirus, COVID-19, value of human life, net gross domestic product, Turkey

## Abstract

**Background: ** This study aimed to appraise the monetary value of human life losses associated with COVID-19 in Turkey. To our knowledge, it is the first study in Turkey to value human life losses associated with COVID-19.

**Methods: **A human capital approach (HCA) model was applied to estimate the total monetary value of the 4,807 human lives lost in Turkey (TMVHL) from COVID-19 by 15 June 2020. The TMVHL equals the sum of monetary values of human lives lost (MVHL) across nine age groups. The MVHL accruing to each age group is the sum of the product of discount factor, years of life lost, net GDP per capita, and the number of COVID-19 deaths in an age group. The HCA model was re-calculated five times assuming discount rates of 3%, 5%, and 10% with a national life expectancy of 78.45 years; and the world highest life expectancy of 87.1 years and global life expectancy of 72 years with 3% discount rate.

**Results: **The 4807 human life losses from COVID-19 had a TMVHL of Int$1,098,469,122; and a mean of Int$228,514 per human life. Reanalysis with 5% and 10% discount rates, holding national life expectancy constant, reduced the TMVHL by Int$167,248,319 (15.2%) and Int$ 429,887,379 (39%), respectively. Application of the global life expectancy reduced the TMVHL by 36.4%, and use of world highest life expectancy increased TMVHL by 69%. However, the HCA captures only the economic production losses incurred as a result of years of life lost. It ignores non-market contributions to social welfare and the adverse effects of economic activities.

**Conclusions: **Additional investment is needed to bridge the persisting gaps in International Health Regulations capacities, Universal Health Coverage, and safely managed water and sanitation services.

## Introduction

Turkey has a population of 84.040 million people; a total gross domestic product (GDP) of International Dollars (Int$) 2,464.61 billion; a GDP per capita of Int$ 29,326.503 in 2020 ^1^; a human development index (HDI) of 0.806 in 2018 ^2^; an inequality-adjusted HDI of 0.675 in 2018 ^2^; and a Gini Coefficient of 41.9 in 2017 ^2^. National income share held by the poorest 40% is 15.6% compared to 32.1% held by the wealthiest 10% and 23.1% held by the wealthiest 1% in 2017 ^2^. The real GDP growth is predicted to decline by 5.0% during 2020 due to COVID-19 pandemic ^3^.

As of 15 June 2020, Turkey had notified a total of 178,239 coronavirus disease 2019 (COVID-19) cases, which included 4,807 deaths, 151,417 recoveries, and 22,015 active cases ^4^. There were a 2,114 total cases per million population; 57 deaths per million population; and 31,225 COVID-19 tests per million population. The rate of COVID-19 transmission may hinge on the strength of International Health Regulations (IHR) core capacities ^5^, the extent of universal health coverage (UHC) ^6^, and population coverage of safely managed water and sanitation services.

IHR core capacity refers to the minimum core public health capability to detect, assess, notify and report events, and respond promptly and effectively to public health risks and public health emergencies of international concern ^5^. There are 13 IHR core capacities, including national legislation, policy and financing; coordination and national focal point functions; surveillance; response; preparedness; risk communication; human resources; laboratory; points of entry; and the four IHR potential hazards (chemical, zoonotic, food safety, and radio-nuclear events). Each of the core capacities is assessed on a scale of ranging from 0% (non-existent) to 100% (optimal/target). The national IHR capacity score is an average of the 13 core capacities ^7^.

In 2017, Turkey had an average IHR core capacity score of 77% ^8^, implying an overall gap of 23%, i.e. the difference between the optimal (100%) and actual Turkey’s capacity of 77%. The IHR capacity components of legislation and financing, points of entry, and zoonotic events and human-animal interface had a score of 100, implying optimal target capacities ^9^. The scores for the laboratory was 93; surveillance was 90; health service provision was 87; the chemical events were 80; coordination and national focal point functions was 70; human resources were 60; national health emergency framework was 60; food safety was 60; radiation emergencies were 60 and; risk communication was 40 ^9^. These scores imply gaps of 7, 10, 13, 20, 30, 40, 40, 40, 40, and 60 in the IHR components of laboratory, surveillance, health service provision, chemical events, coordination and national focal point functions, human resources, national health emergency framework, food safety, radiation emergencies, and risk communication.

The United Nations (UN) Sustainable Development Goal 3.8 is about achieving UHC, including access to high-quality essential healthcare services for all ^10^. The WHO and World Bank UHC index, measured on a scale of 0% to 100%, is a measure of average proportion of people in need receiving reproductive, maternal, new-born and child health services; infectious diseases (including COVID-19) prevention and management services; and non-communicable diseases prevention and control services ^6^. According to WHO ^8^, the UHC index for Turkey is 74%, signifying that 26% of people in need do not receive high quality essential health services. Approximately 2,689,280 (3.2%) of the population has health expenditures that are over 10% of total household income, implying a high risk of impoverishment ^8^. The Turkish Government should assure access to COVID-19 prevention, testing, treatment and palliative services, especially for this vulnerable segment of the population.

About 840,400 (1%) of the population use unimproved drinking water sources ^11^, and 29,414,000 (35%) of the population have no access to safely managed sanitation services ^8^. These people have difficulty practising personal hygiene measures recommended by WHO for the prevention and control of COVID-19 ^12^.

Monetary valuation of human life is useful in quantifying the size of disease burden in dollar terms ^13^, building a justification for intervention programmes and research ^13^ and advocacy for increased investments ^14^ to bridge gaps in IHR capacities, UHC, and safely managed water and sanitation services. There is a paucity of literature on the valuation of human life losses associated with COVID-19 ^15– 17^. This study appraised the monetary value of human life losses associated with COVID-19 in Turkey as of 15 June 2020.

## Methods

### Empirical framework for monetary valuation of human life

This study replicates the human capital approach (HCA) methodology proposed by Weisbrod in 1961 ^18^ and applied recently in China ^15^, USA ^16^, and Iran ^17^ to estimate the monetary value of 4,792 human life losses associated with COVID-19 in Turkey as of 15 June 2020. The premature death of a person from COVID-19 (or any other cause) results in potentially productive years of life lost (YLL), which is the difference between the average age of onset of death and the mean life expectancy at birth for Turkey ^15^. The total monetary value of human life losses (TMVHL _Turkey_) associated with COVID-19 in Turkey equals the addition of monetary values of human lives lost across age groups 1=0–9 years, 2=10–19 years, 3=20–29 years, 4=30–39 years, 5=40–49 years, 6=50–59 years, 7=60–69 years, 8=70–79 years, and 9=80 years and above. Formally:

$${\text{TMVHL}}_{\text{Turkey}}=\sum_{k=1}^{k=9}{\text{MVHL}}_{k}\;\text{(1)}$$

The monetary value for human life losses accruing to each *k ^th^* age group (MVHL _*k*=1,.,9_) is the sum of the product of discount factor, years of life lost, net GDP per capita, and the number of COVID-19 deaths in an age group ^15^. Algebraically:

$${\text{MVHL}}_{k}=\sum_{\beta =1}^{\beta =n}\left(D_{1})\times (D_{2}-D_{3})\times (D_{4}-D_{5})\times (D_{6}\times D_{7}\right)\;\text{(2)}$$

Where: $\sum_{\beta =1}^{\beta =n}$ is a summation from the 1 ^st^ to the *n* ^th^ year of life lost; *D*_1_ is the discount factor (1/(1+ *r*) ^*n*^) where *r* is the discount rate of 3%; *D*_2_ is the mean life expectancy at birth of Turkey; *D*_3_ is the mean age of onset of death in k ^th^ age group; *D*_4_ is the GDP per capita for Turkey; *D*_5_ is the current health expenditure per person in Turkey; *D*_6_ is the total number of COVID-19 deaths in Turkey as of 15 June 2020; *D*_7_ is the proportion of COVID-19 deaths borne by k ^th^ age group.

### The sensitivity analysis

According to Thabane *et al.* ^19^, sensitivity analysis is a way of assessing the effect of variations in key assumptions on overall conclusions of the study. In this study, a sensitivity analysis was conducted to answer two questions: What would happen to the TMVHL if the discount rate was varied from 3% while holding Turkey’s life expectancy at birth constant? What would happen to the TMVHL if the life expectancy at birth was varied while holding discount rate constant? A one-way sensitivity analysis was undertaken, where each parameter was varied at a time to investigate the impact on TMVHL ^20^. Thus, the economic model was re-calculated five times. First, using a discount rate of 3% and Turkey’s national life expectancy at birth. Second, using a discount rate of 5% and Turkey’s national life expectancy at birth. Third, using a 10% discount rate and Turkey’s national life expectancy at birth ^21^. Fourth, assuming the average global life expectancy of 72 years ^8^ and a 3% discount rate. Fifth, assuming the average global life expectancy of 87.1 years ^8^ and a 3% discount rate. The discount rates and global and world highest life expectancies were chosen because they are frequently used in past economic studies ^14– 17, 22– 24^.

### Data and data sources

Table 1 contains the data and data sources used to estimate the economic model. The model was estimated using Microsoft Excel Software.

**Table 1.** Data and data sources for the Turkey analysis.

Variable	Data	Data sources
Discount rate (r)	3%, 5%, 10%	Kirigia and Muthuri ^15, 16^, Kirigia Muthuri and Muthuri ^17^.
Mean life expectancy at birth (LEB) ( *D*_2_)	Turkey LEB = 78.45 years ^21^; global LEB = 72 years ^8^; Japanese Females LEB (world’s highest) = 87.1 years ^8^.	Worldometer database ^21^ and WHO world health statistics report 2020 ^8^.
Mean age of onset of death in k ^th^ age group ( *D*_3_)	0–9 years = (0+9)/2=4.5 years; 10–19 years = 14.5 years; 20–29 years = 24.5 years; 30–39 years = 34.5 years ; 40–49 years = 44.5 years; 50–59 years =54.5 years; 60–69 years = 64.5 years; 70–79 years = 74.5 years; 80 years and above = 80 years.	Authors estimates from age groups in Verity *et al.* ^25^.
GDP per capita for Turkey ( *D*_4_)	Int$ 29,326.503	International Monetary Fund (IMF) World Economic Outlook Database ^1^.
Current health expenditure per person in Turkey ( *D*_5_)	Int$ 1,181	WHO Global Health Expenditure Database ^26^.
Total number of COVID- 19 deaths in Turkey as of 15 June 2020 ( *D*_6_)	4,807	Worldometer database ^4^.
Proportion of COVID-19 deaths per age group in Turkey	0–9 years= 0; 10–19 years = 0.000977517; 20–29 years = 0.00684262; 30–39 = 0.017595308; 40–49 years = 0.03714565; 50–59 years = 0.127077224; 60–69 years = 0.302052786; 70–79 years = 0.304985337; and 80 and older = 0.203323558.	Turkey’s COVID-19 deaths are no disaggregated by age. Thus, we used age break-down from Verity *et al.* ^25^.

## Results

### Analysis assuming Turkey’s mean life expectancy of 78.45 years and a 3% discount rate

As depicted in Table 2, the 4807 human life losses from COVID-19 had a total monetary value of Int$ 1,098,469,122; and a mean of Int$228,514 per human life.

**Table 2.** The total and average discounted monetary value of human life losses from COVID-19 in Turkey – assuming the national mean life expectancy of 78.45 years and a 3% discount rate (in 2020 Int$).

Age group in years	Monetary value of human lives lost at 3% discount rate (Int$)	Average monetary value per human life lost in an age group (Int$)
0–9 ^*^	0	0
10–19	3,743,634	796,700
20–29	24,604,939	748,042
30–39	57,738,871	682,649
40–49	106,200,936	594,766
50–59	291,172,234	476,659
60–69	461,629,419	317,934
70–79	153,379,089	104,620
80 & over ^**^	0	-
**TOTAL**	1,098,469,122	228,514

Note: ^*^Monetary value for 0-9-year-olds was zero because there was no death in that age group. ^**^Monetary value for 80-year-olds and above was zero due to zero years of life lost at the age above the mean life expectancy of Turkey.

Of the TMVHL, 0% was held by 0–9-year-olds, 0.3% by 10–19-year-olds, 2.2% by 20–29-year-olds, 5.3% by 30–39-year-olds, 9.7% by 40–49-year-olds, 26.5% by 50–59-year-olds, 42.0% by 60–69-year-olds, 14.0% by 70–79-years-olds, and 0.0% by 80-year-olds and above. The persons aged between 30 and 69 years accounted for 83.5% of the TMVHL. The monetary value per human life lost among 10–19-year-olds was 8-fold that of 70–79-year-olds.

### The sensitivity of TMVHL to variations in the discount rate

Table 3 presents the TMVHL estimated alternately at 5% and 10% while holding the national life expectancy at 78.45 years.

**Table 3.** The discounted monetary value of human life losses from COVID-19 in Turkey – assuming 5% and 10% discount rates (in 2020 Int$).

Age group, years	Monetary value of human life lost at 5% discount rate (Int$)	Monetary value of human life lost at 10% discount rate (Int$)
0–9	-	-
10–19	2,528,573	1,319,569
20–29	17,187,151	9,203,888
30–39	42,047,376	23,446,394
40–49	81,379,654	48,289,208
50–59	237,239,607	154,474,417
60–69	404,521,448	301,049,720
70–79	146,316,993	130,798,549
80 & above	-	-
**TOTAL**	931,220,803	668,581,743
**Monetary** **value per** **human life**	**193,722**	**139,085**

Reanalysis of the economic model with 5% discount rate, holding national life expectancy constant, reduced the TMVHL by Int$167,248,319 (15.2%); and the mean value per human life by Int$34,793. The application of a discount rate of 10% shrank the TMVHL by Int$ 429,887,379 (39%), and the mean monetary value per human life by Int$89,429.

### The sensitivity of TMVHL to variations in mean life expectancy holding discount rate contact at 3%

Table 4 depicts the TMVHL of the human life losses from COVID-19 estimated consecutively assuming the mean global life expectancy of 72 years and the world highest life expectancy of 87.1 years.

**Table 4.** The discounted monetary value of human life losses from COVID-19 in Turkey – assuming mean global and world's highest life expectancies (in 2020 Int$).

Age group in years	The monetary value of human life lost at 3% discount rate and mean global life expectancy of 72 years (Int$)	The monetary value of human life lost at 3% discount rate and the world highest life expectancy of 87.1 years (Int$)
0–9	-	-
10–19	3,614,624	3,898,924
20–29	23,391,290	26,065,823
30–39	53,544,762	62,787,374
40–49	94,301,593	120,524,330
50–59	236,463,710	357,025,594
60–69	286,869,285	671,990,460
70–79	-	438,831,473
80 & above	-	171,387,289
**TOTAL**	698,185,265	1,852,511,267
**Monetary** **value per** **human life**	**145,243**	**385,378**

Re-calculation of the economic model with the global life expectancy of 72 years (which is about six years less than national life expectancy), holding discount rate constant at 3%, reduced the TMVHL by Int$400,283,857 (36.4%), and the mean value per human life by Int$83,271. Application of the world highest life expectancy of 87.1 years, while holding discount rate of 3% constant, grew TMVHL by Int$ 754,042,145 (69%), and mean monetary value per human life by Int$ 156,863.

## Discussion

### Key findings and implications

The key findings are as follows:

- The 4807 human life losses from COVID-19 had a TMVHL of Int$1,098,469,122; and a mean of Int$228,514 per human life.
- Reanalysis with 5% and 10% discount rates, holding national life expectancy constant, reduced the TMVHL by Int$167,248,319 (15.2%) and Int$ 429,887,379 (39%), respectively.
- Application of the global life expectancy of 72 years reduced the TMVHL by Int$ 400,283,857 (36.4%) and use of world highest life expectancy of 87.1 years increased TMVHL by Int$ 754,042,145 (69%).

The total monetary value of human life losses associated with COVID-19 was equivalent to 0.045% of the total GDP for Turkey. The mean monetary value per human life lost was eight times the size of the GDP per capita for Turkey in 2020. The magnitude of TMVHL will continue growing as the pandemic persists.

The study found that use of higher discount rates produced lower TMVHL, which is consistent with past economic studies ^15– 17, 27, 28^. Furthermore, the analysis has revealed that TMVHL is quite sensitive to both the sizes of the discount rate used to convert the monetary value of future YLL into their present values; and the magnitude of the life expectancy at birth. The latter determines the number of YLL.

### Comparison with similar studies in other countries

As alluded earlier, globally, there is a dearth of literature on the valuation of human life losses associated with COVID-19. The mean of Int$228,514 per human life lost from COVID-19 was lower than Int$ 470,798 in Spain ^28^, Int$ 356,203 in China ^15^ and Int$ 292,889 in USA ^16^ but higher than Int$ 103,090 in the Islamic Republic of Iran ^17^. The findings from Spain, China, USA and Iran studies are comparable to the current study in Turkey because they employed a similar methodology, i.e. the human capital approach.

### Study limitations

This study has some weaknesses. First, it employs the human capital approach to value human life losses associated with COVID-19. As Mooney ^29^ explains “… *equates the value of life with the value of livelihood. Clearly, there are major problems with this omission of non-work values, particularly as it leaves the valuation of pensioners and many women at worst at zero*…” (p. 65). The health services have other objectives apart from returning the sick to work. For instance, enabling people to realise their rights to health and life ^30^, enjoy the leisure and flourish ^31^, and fulfil non-economic societal roles (e.g. sports, socialising, religious activities, marital affairs, nurturing children, diffusing tacit knowledge, community participation). The human capital approach captures only the economic production losses incurred as a result of YLL.

Second, while GDP per capita is a good indicator of economic activity in a country, it ignores non-market contributions to social welfare; distribution of income and wealth; quality of life (or wellbeing); and adverse effects (including pollution) of the economic production process on the environment ^22^.

Third, the study did not capture the multi-sectoral production inputs expended on COVID-19 prevention, diagnosis, quarantine, contact tracing, treatment, mental health care, rehabilitation, post-mortem, and burials ^15– 17, 28^.

Fourth, although there is consensus that future monetary values of YLL ought to be adjusted to their present values, there is no consensus in the health economics literature on the discount rate to be used ^32, 33^. In this study, we chose to use a discount rate of 3% because of extensive use in health-related economic studies ^15– 17, 23, 24, 28^. As mentioned earlier, due to the uncertainty surrounding the choice of a discount rate, a sensitivity analysis was conducted using 5% and 10% discount rates to test the robustness of the TMVHL ^15– 17, 28^.

### Suggestions for further research

The following three economic studies would be useful to the health development policymakers in Turkey:

- Cost of multi-sectoral resources invested in COVID-19 prevention and control measures.
- Estimation of resources needed to bridge the persisting gaps in IHR capacities, UHC, and safely managed water and sanitation services.
- Full economic evaluations (including cost-benefit, cost-utility, and cost-effectiveness analyses) of alternative options related to COVID-19 prevention (e.g. lockdown, physical distancing, personal hygiene), diagnosis (testing), quarantine, contact tracing, treatment, mental health care, and rehabilitation ^34, 35^.

## Conclusion

The average monetary value per human life loss associated with COVID-19 was eight-fold that of the GDP per capita for Turkey in 2020. Thus, COVID-19 pandemic is imposing a substantive burden on both population health but also the economy of Turkey. There is an urgent need for the country to invest more in health-related sectors to bridge the persisting gaps in IHR core capacities, UHC, and safely managed water and sanitation services to eradicate the ongoing COVID-19 pandemic and mitigate future public health emergencies.

## Data availability

### Source data

The economic model was estimated using data from the following sources:

- The Turkey life expectancy at birth data from the Worldometer database ^21^: https://www.worldometers.info/demographics/life-expectancy/#countries-ranked-by-life-expectancy.
- The global meant life expectancy and the world highest life expectancy from the WHO World Health Statistics 2020 ^8^ or from the Global Health Observatory: https://www.who.int/gho/publications/world_health_statistics/en/.
- Per capita GDP for Turkey (in International Dollars) from the International Monetary Fund (IMF) World Economic Outlook Database ^1^: https://www.imf.org/external/pubs/ft/weo/2019/02/weodata/index.aspx.
- Current health expenditure per person in Turkey from the WHO Global Health Expenditure Database ^26^: https://apps.who.int/nha/database/ViewData/Indicators/en.
- Total number of COVID-19 deaths in Turkey as of 15 June 2020 from the Worldometer database ^4^: https://www.worldometers.info/coronavirus/country/Turkey/.
