# Supplementary material for: The monetary value of human life losses associated with COVID-19 in Turkey
Source: Emerald Open Res. 2020 Jul 8;2:44. doi: 10.35241/emeraldopenres.13822.1 (PMC7390608; doi:10.35241/emeraldopenres.13822.1)
Supplement: Peer review history

# Peer review: Reviewer response for version 1

**DOI:** 10.21956/emeraldopenres.14900.r26977

**Published:** 2020-07-27

**Authors:** Claudio Politi

**Affiliations:** 1 Polio Transition Team, World Health Organization (WHO), Geneva, Switzerland

I recommend to double check all the figures cited in the text and those reported in the tables. There are several inconsistencies e.g. Int$ 167,248,319 and Int$ 429,887,379 cited in the abstract and in the sensitivity analysis but those are different from the values reported in tables. Also the figures discussed in the section key findings and implications do not correspond to the values reported in the tables.

 I would suggest to discuss further the results obtained for the age-classes above 80yrs, that is 0 Int$ in the baseline and also for the age-classes above 70 yrs in the sensitivity analysis.

 Furthermore it would be useful to elaborate more the comparison with similar studies in other countries, by explaining the variables that explain the differences and the limitations of the methodology for policy making.

Is the work clearly and accurately presented and does it cite the current literature?

Yes

If applicable, is the statistical analysis and its interpretation appropriate?

Yes

Are all the source data underlying the results available to ensure full reproducibility?

Yes

Is the study design appropriate and is the work technically sound?

Yes

Are the conclusions drawn adequately supported by the results?

Yes

Are sufficient details of methods and analysis provided to allow replication by others?

Yes

Reviewer Expertise:

Health Financing and Immunization

I confirm that I have read this submission and believe that I have an appropriate level of expertise to confirm that it is of an acceptable scientific standard.

# Peer review: Reviewer response for version 1

**DOI:** 10.21956/emeraldopenres.14900.r26975

**Published:** 2020-07-21

**Authors:** Eyob Zere Asbu

**Affiliations:** 1 Health System Financing Division, Department of Health, Abu Dhabi, United Arab Emirates

This timely and informative study aims to assess the monetary value of human losses in Turkey attributed to the COVID-19 pandemic. The method used is the human “capital approach” (HCA). 

 The authors have planned and executed this study well and demonstrated their knowledge and skills in relation to cost of illness studies, particularly (HCA).

 It would be worthwhile if the authors would consider the following minor comments:

1. Have a separate brief country profile comprising all the statistical data in the introduction section. This will leave the introduction to state and elaborate the problem, objectives and significance of the study.
2. Introduction section, Paragraph 1: Instead of Gini coefficient, state as “Gini index”, as it is expressed in the scale of 0-100.
3. The issues related to IHR and UHC discussed in the introduction section may preferably be included in the country profile (the statistical aspects such as IHR core capacity score and UHC index) and the link with COVID-19 transmission be reserved for the discussion section.
4. Page 4 - Sensitivity analysis, 5th line from last: “... assuming the average global life expectancy of 87.1 year...” - This should be the global max life expectancy. Please revisit.

Others:

- Apart from providing Turkey’s GDP per capital, it would also be more informative if the income status of the country is mentioned (per World Bank’s classification)
- Providing the GDP per capital at average exchange rate would be useful. Alternatively if the PPP conversion factor is provided, which could be of use to apply to all figures expressed in Int$.

Is the work clearly and accurately presented and does it cite the current literature?

Yes

If applicable, is the statistical analysis and its interpretation appropriate?

Yes

Are all the source data underlying the results available to ensure full reproducibility?

Yes

Is the study design appropriate and is the work technically sound?

Yes

Are the conclusions drawn adequately supported by the results?

Yes

Are sufficient details of methods and analysis provided to allow replication by others?

Yes

Reviewer Expertise:

Health economics, health systems and public health

I confirm that I have read this submission and believe that I have an appropriate level of expertise to confirm that it is of an acceptable scientific standard.
